# Books and Pamphlets Received

**Published:** 1888-12

**Authors:** 


### BOOKS AND PAMPHLETS RECEIVED.

*Second Annual Report of the State Board of Health*, and vital statistics of the Commonwealth of Pennsylvania. Transmitted to the Governor Dec. 1, 1886. Harrisburg. Edwin R. Meyers. 1887.

This is, we believe, the largest and most elaborate report published in any of the states relating to sanitary subjects. It contains much practical and valuable information connected with the "sanitary condition of cities and towns," "epidemics and special sources of diseases," "quuarantine and disinfections," numerous papers on hygienic topics, "geographical disinfection of consumption and malarial diseases," "laws and decisions relating to public health," moruary tables, etc., etc. Neatly gotten out in cloth and containing 1058 large oc. pages.

*Treatise on the diseases of women* for the use of students and practitioners. By Alexander J. C. Skene, M. D, Professor of Gynecology in the Long Island College Hospital, Brooklyn, N. Y., formerly Professor of Gynecology in the New York Post Graduate Medical School, Gynecologist to the Long Island College Hospital, President of the American Gynecological Society 1887, Corresponding member of the British, Boston and Detroit Gynecological Societies, Fellow of the New York Academy of Medicine, etc., etc. With 251 engravings and 9 chromo-lithographs. New York. D. Appleton & Co. 1888.

In the above we have a very able as well as a large and comprehensive work of 966 octavo pages, embracing every important subject connected with gynecology. The subjects described are illustrated with typical and complicated cases many of which are drawn from the practice of the author. Space has not been wasted on the discussion of unsettled questions, and practical rather than theoretical views have been given. The type is bold and neat, and the illustrations are admirable. The work is adapted to the wants both of the student and practitioner, and he has presented in operative procedures the latest and most improved methods. It is certainly a very instructive and desirable work on Gynecology. For the present we are informed that it is sold only by subscription. Competent canvassers can secure agencies by applying to Mr. T. K. Oglesby, manager for D. Appleton & Co., Atlanta, Ga.

*Table Talk, January Number*, 1888—Devoted to the needs of American households. Published in Philadelphia. Forty pages, double column. Interesting, varied and instructive. Terms $1.00 per annnm.

*Address on Rhinology*.—The President's address before the American Rhinological Association. By Carl H. von Klein, A. M., M. D., of Dayton, Ohio. Delivered at the annual meeting, Cincinnati, Ohio, September 12, 13 and 14, 1888.

The October number of *The International Journal of Surgery and Antiseptics* contains an excellent likeness of the late Dr. C. R. Agnew, of New York. The subscription of the Journal is $1.00 a year. Single copy 30 cents. Dr. F. King, Manager, P. O. Box 587, New York.

